# Factors associated with common and atypical chromosome abnormalities after positive combined first-trimester screening in Chinese women: a retrospective cohort study

**DOI:** 10.1186/s12884-019-2205-y

**Published:** 2019-02-04

**Authors:** Annisa Mak, Helena Lee, C. F. Poon, S. L. Kwok, Teresa Ma, K. Y. K. Chan, Anita Kan, Mary Tang, K. Y. Leung

**Affiliations:** 1 Department of Obstetrics and Gynaecology, Queen Elizabeth Hospital, 30 Gascoigne Road, Hong Kong, SAR China; 2 Department of Obstetrics and Gynaecology, Queen Mary Hospital, The University of Hong Kong, 102 Pokfulam Road, Hong Kong, SAR China

**Keywords:** Cell-free DNA screening, Non-invasive prenatal testing, Risk of atypical aneuploidies, Detection rate, Aneuploidy screening, Down syndrome

## Abstract

**Background:**

When cell-free DNA (cfDNA) testing is used as a secondary screening tool following combined first-trimester screening (cFTS), cFTS is used to estimate the prior risk for chromosome abnormalities. This study aimed to assess the factors that are associated with common and atypical abnormalities following cFTS, including cFTS risk, advanced maternal age, increased nuchal translucency (NT) ≥3.5 mm, and abnormal levels of serum markers.

**Methods:**

We reviewed a historical cohort of 1855 Chinese women carrying singleton pregnancies with a positive cFTS [at a threshold of 1:250 for trisomy (T) 21 or 1:180 for T18] in one public hospital over a five-year period. All chromosome abnormalities were confirmed by invasive prenatal diagnosis (IPD) with karyotyping, with or without array comparative genomic hybridization. Using multivariable binary logistic regression analysis, we determined the parameters that were associated with common and atypical abnormalities.

**Results:**

Overall, the prevalence of common and atypical abnormalities was 6.2 and 1.2%, respectively, and the prevalence increased with the risk of T21 by cFTS. In pregnancies with a risk of T21 > 1 in 100, a high risk of both T21 and T18, an increased NT, or a pregnancy-associated plasma A (PAPP-A) level <  0.2 multiple of medians (MoM), the prevalence of common abnormalities was 12.2, 64.7, 25.5 and 33.8%, respectively, while that of atypical abnormalities was 1.6, 3.9, 4.2, and 7.4%, respectively. In the multivariable binary logistic regression analysis, out of these four factors, only two (increased NT and PAPP_A <  0.2 MoM) were significant predictors of common and atypical abnormalities, respectively. Of all positive cFTS pregnancies, 50.4% did not have any of these four factors, and the prevalence of common and atypical abnormalities was 1.1 and 0.6%, respectively. There were three atypical abnormalities, all of which were mosaicism, and they were detected among women with IPD alone. The ages of these women were ≥ 35 years. All three pregnancies were continued after proper counseling. After giving birth, only one child had mild abnormalities, while the other two were phenotypically normal.

**Conclusions:**

Our study identified factors associated with common and atypical abnormalities after cFTS. These factors can be used to estimate the prior risk for these abnormalities to help with post-cFTS counseling in terms of choosing between cfDNA testing and IPD.

## Background

A recent meta-analysis showed that non-invasive prenatal testing (NIPT) using cell-free DNA (cfDNA) in singleton pregnancies could detect > 99% of fetuses with trisomy 21 at a false positive rate (FPR) of 0.04% [1]. Short of offering women of any background risk of cfDNA testing as a primary screening tool [2], which is relatively expensive, universal combined first trimester screening (cFTS) is still an acceptable method of prenatal screening for trisomy 21 [3–5] because it can achieve a sensitivity of approximately 93% [6] and allows for the opportunity of an early detection of fetal structural abnormalities during nuchal translucency (NT) scanning.

Following a positive cFTS, cfDNA testing can be used as a secondary screening tool [3–5] because cfDNA testing can detect > 99% of fetuses with trisomy 21, 98% of those with trisomy 18 and 99% of those with trisomy 13 at a combined FPR of 0.13%. It seems that cfDNA testing can also detect > 95% of fetuses with sex chromosome aneuploidies (SCA) at an FPR of < 1% according to small scale studies [1]. The use of cfDNA testing was accompanied by a significant decline in the rate of invasive prenatal diagnosis (IPD) following positive cFTS results [7–10] and a concomitant decrease in the potential risk of procedure-related fetal losses, albeit small (0.11–0.22%) according to a recent meta-analysis [11]. However, approximately 17–50% of abnormal chromosomes, including clinically relevant atypical abnormal karyotypes, may not be detected by targeted cfDNA testing focusing on common aneuploidies compared to IPD with chromosome microarray (CMA), which can enable the detection of submicroscopic duplications and deletions [12–14].

While cfDNA testing is used as a secondary screening tool, it is desirable to include a protocol in which women with very high risk are offered IPD and those with intermediate risk are offered cfDNA testing [4]. It is well known that IPD should be offered to women with pregnancies with an increased fetal nuchal translucency (NT) thickness (≥ 3.5 mm) or an ultrasound-detected abnormality because cfDNA testing may miss atypical aneuploidies at a rate of up to 8% [15]. In addition, recent studies on Danish and Australian populations suggest that the worry of missing atypical abnormalities (other than trisomies 13, 18, and 21 or SCA) can be reduced by offering IPD to pregnancies with a high trisomy 21 risk (> 1 in 100) or a low pregnancy associated plasma protein-A (PAPP-A) level (< 0.2 MoM) [12, 16].

The main objectives of our present study are to determine which factors, among cFTS risk, advanced maternal age, increased nuchal translucency (NT) ≥3.5 mm, and abnormal levels of serum markers, are associated with common and atypical abnormalities. If an individual’s risk of common and atypical abnormalities can be estimated by considering various parameters of cFTS, this information would be useful for counseling before and after cfDNA testing.

## Methods

A retrospective cohort study was conducted on all women who had a singleton pregnancy and a prenatal screening for Down syndrome at Queen Elizabeth Hospital (QEH) in Hong Kong between August 2011 and July 2016. QEH is a public hospital with approximately 6000 deliveries a year, and the majority of the pregnant women are Chinese. This study was approved by the Research Ethics Committee of Kowloon Central/Kowloon East Cluster in Hong Kong, and patients’ consents were not required because this was a retrospective study. Furthermore, all the participants were anonymous.

Universal cFTS, involving an assessment of the NT, free β-human chorionic gonadotropin (fβ-hCG) and PAPP-A, was offered between 11 and 13 weeks and 6 days. The gestational age was determined by an ultrasonographic measurement of the crown rump length shortly after the first antenatal visit.

Women with a screening result for trisomy 21 of 1 in 250 or higher or with a screening result for trisomy 18 of 1 in 180 or higher were reported as ‘high risk’. These women were counseled by the maternal fetal medicine team of QEH on different options, including chorionic villus sampling (CVS), amniocentesis, cfDNA testing or no further testing. Women with a fetal NT ≥ 3.5 mm or with structural abnormalities were advised to undergo IPD rather than cfDNA testing, given that the latter may miss atypical aneuploidies at a rate of 8% [15]. All women were followed up for a mid-trimester anomaly scan and further counseling, post IPD or cfDNA testing. For those women who underwent cfDNA testing, IPD was offered for a positive cfDNA result, an ultrasound-detected fetal abnormality or a maternal concern.

Karyotype analyses of all the prenatal samples were performed by the Prenatal Diagnosis Laboratory of Tsan Yuk Hospital and Prenatal Genetic Diagnosis Centre at Prince of Wales Hospital, both of which are public prenatal diagnostic laboratories in Hong Kong. The analyses included quantitative fluorescent polymerase chain reaction for rapid aneuploidy detection and G-banded chromosome analyses. Array comparative genomic hybridization (aCGH) or fluorescence in situ hybridization (FISH) was performed for an ultrasound abnormality or for the characterization of a chromosome abnormality. NimbleGen manufactured CGX-135 k oligonucleotide array (Roche Diagnostics GmbH, Germany) was used for the samples analyzed in TYH before April 2014, and, thereafter, Agilent manufactured CGX 60 k oligonucleotide array (Perkin Elmer, USA) was used. Fetal DNA Chip (manufactured by Agilent) was used for the samples processed in PWH.

Commercial cfDNA testing was based on massively parallel sequencing with ‘shotgun’ counting of all cfDNA sequences or ‘targeted’ counting of specific DNA sequences or single nucleotide polymorphisms. The majority of the cfDNA testing were performed in three laboratories. All cfDNA reports included the risks for trisomies 21, 18, and 13 and, recently, the risks of SCA, selected microdeletions or duplications and aneuploidies involving other chromosomes.

cFTS and IPD were publicly funded, while the cfDNA testing and aCGH were self-financed (around $700 USD per test). Pregnancy outcomes were traced by reviewing hospital records or by phone contact for women who delivered outside of this hospital. Chromosomal analyses were performed after birth if it was clinically indicated, such as for the finding of congenital anomalies. We defined balanced translocation as ‘normal’ after the exclusion of microdeletions or duplications by aCGH and counseled women on their reproductive risk in the future. The women were also referred to clinical geneticists for family counseling or testing.

### Statistical analysis

With the use of descriptive statistics, we determined the prevalence of common abnormalities (trisomies 13, 18 and 21 or SCA) and atypical abnormalities (including mosaicism or other abnormalities but excluding balanced translocations). Using the chi-square test, we assessed the prevalence of common and atypical abnormalities by using various known factors, including a maternal age ≥ 45, an NT thickness ≥ 3.5 mm, a risk of trisomy 21 > 1 in 100, a fβ-hCG < 0.2 or ≥ 5.0 multiple of medians (MoM), and a PAPP-A < 0.2 MoM [11]. We then included those significant factors and used a separate multivariable binary logistic regression analysis for the predictions of common and atypical abnormalities. In pregnancies without any of the significant factors, the prevalence of common and atypical abnormalities was compared between women receiving IPD alone and those receiving cfDNA testing.

To assess the extent of missed atypical abnormalities while some women chose cfDNA testing instead of IPD during the study period, we compared the prevalence of chromosome abnormalities between the one-year period before and the five-year period after the introduction of cfDNA testing. The statistical package software SPSS 21.0 (IBM, SPSS, Statistics for Windows, Version 21.0; IBM, Armonk, N.Y., U.S.A.) was used for all analyses.

## Results

During the study period, 33,104 (97.5%) of the 33,952 pregnant women underwent routine Down syndrome screening, 28.9% of whom were aged 35 years or older. There were 2201 (6.7%) positive-screen pregnancies. After excluding 136 multiple pregnancies and 210 s trimester screenings, we studied the remaining 1855 women who had a positive cFTS, 95.8% of whom were Chinese, 752 of whom underwent a cfDNA testing, 986 of whom had IPD alone, and 117 of whom had no further testing at our hospital or at other places (Table 1). The prevalence of common chromosomal abnormalities was 6.2% (115/1855) and that of atypical abnormalities was 1.2% (22/1855) in the positive cFTS pregnancies (Table 1). The screening results of these 22 patients with atypical abnormalities are detailed in Table 2.

**Table 1:** Prevalence of chromosomal abnormalities after cell-free DNA (cfDNA) testing, invasive prenatal diagnosis (IPD) alone, and birth in singleton pregnancies following positive conventional screening results; *n* (%)

	cfDNAtesting *n* = 752	IPD alone *n* = 986	No further test *n* = 117	Total
cfDNA testing	752	0		
Abnormal results	24 (3.2%)	–		
IPD or karyotyping after birth^c^	34 (4.5%)^a^	986(100.0%)	7 (6.0%)^c^	
Chromosome abnormalities	21 (2.8%)	120 (12.2%)	7 (6.0%)	148
Trisomy 21	14 (1.9%)	59 (6.0%)	2 (1.7%)	75
Trisomy 18 or 13	3 (0.4%)	22 (2.2%)	4 (3.4%)	29
Sex chromosome aneuploidy	2 (0.3%)	9 (0.9%)	–	11
Mosaicism	0 (0%)	10 (1.0%)	–	10
Other atypical abnormalities	1 (0.1%)	10 (1.0%)	1 (0.9%)^d^	12
Balanced translocation	0 (0%)	10 (1.0%)	–	10
Unknown^b^	1 (0.1%)	0 (0%)	–	1

^a^Of 34 IPD, 24 were performed for abnormal cfDNA testing and 10 for anxiety, despite a negative cfDNA testing and normal scan findings.

^b^pregnancy was terminated at another hospital.

^c^Karyotyping (a) after birth in the group of women who declined further testing because they would keep their pregnancies anyway or worried about the IPD-related risk of miscarriage or (b) after miscarriage.

^d^Karyotyping of a placental sample after spontaneous miscarriage showed 47,XX,+mar

**Table 2:** Twenty-two patients with atypical chromosome abnormalities and their combined first trimester screening results

	Chromosome abnormalities	T21 risk (1:x)	T18 risk (1:x)	PAPP-A MoM	FB-hCG MoM	NT (mm)	NT MoM
1	46,X,+mar	10	970	.34	.78	12.60	7.15
2	46,XX,del(13)(q13q31)dn	240	5900	1.64	.88	6.40	4.12
3	arr[hg19]4p16.3p14(73,000-36,541,871)×1	2	770	1.21	2.77	5.50	2.88
4	47,XX,+mar.ish i(9)(p10)(wcp9+)dn	120	180	.63	.24	5.50	3.89
5	arr[hg19]6p12.3(46,449,532-47,682,004)×1 mat	6	2700	1.25	1.22	4.90	2.97
6	47,XX,+ 20	140	27,000	1.06	1.76	2.50	1.40
7	45,XY,rob(13;14)(q10;q10)	230	100,000	1.63	4.07	2.50	1.39
8	47,XY,+ 22	40	92,000	.21	2.05	2.20	1.22
9	46,XX,del(13)(q32)dn	90	10,000	.21	.96	1.90	1.21
10	45,XX,rob(13;14)(q10;q10)mat	170	100,000	.19	1.52	1.60	1.08
11	47,XX,+ 22	11	2800	.09	1.67	1.40	.91
12	46,XY,r(21)p(11.2q22.3)dn	210	100,000	.45	1.47	2.10	1.20
13	mos 47,XX,+ 22[20]/46,XX [10]	2	320	.11	1.74	4.70	3.13
14	mos 47,XX,+del(2) (q11.2) [3]/46,XX [27]	3	130	1.00	.68	4.60	2.87
15	mos 47,XY,+r(18)dn [18]/46,XY [12]	220	10,000	.43	.92	2.10	1.14
16	mos 47,XX,+ 16[18]/46,XX [12]	14	16,000	.18	1.45	1.90	1.20
17	mos 47,XY,+8[4]/46,XY[27]dn	30	100,000	.25	3.46	1.70	1.01
18	mos 47,XX,+mar dn [11]/46,XX [19]	110	100,000	.33	2.18	1.40	.82
19	mos 47,XX,+ 16[8]/46,XX [14]	6	51,000	.12	2.76	1.40	.90
20	mos 45,X [28]/46,X,r(Y)[2]dn	20	6000	1.14	1.03	10.30	5.78
21	mos 45,X [20]/46,XX [20]	11	16,000	1.45	2.98	4.10	2.77
22	mos 45,X [5]/46,XX[55]	180	100,000	.35	1.88	1.80	1.06

Risk of trisomy 21 (T21), risk of trisomy 18 (T18), nuchal translucency (NT), pregnancy associated plasma protein A (PAPP-A), free β-human chorionic gonadotropin (fβ-hCG), and multiple of medians (MoM)

### Factors affecting the prevalence of common and atypical abnormalities

**Fig. 1:**
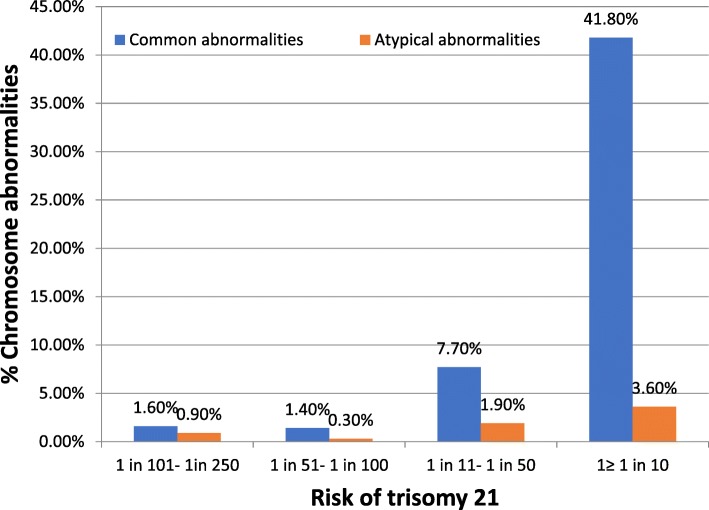
The prevalence of common abnormalities (trisomies 21, 18, and 13 and sex chromosome aneuploidies) and atypical (or other) abnormalities by the risk of trisomy 21 after combined first trimester screening

In general, the prevalence of common and atypical abnormalities increased with an increased risk of trisomy 21 by cFTS (*p* < 0.001), and it increased from 1.6 and 0.9% to 41.8 and 3.6%, respectively, when the cFTS risk was between 1 in 101 and 1 in 250 and when the cFTS risk was ≥1 in 10, respectively (Fig. 1).

The prevalence of common or atypical abnormalities in pregnancies with a risk of trisomy 21 > 1 in 100, a high risk of both trisomy 21 and trisomy 18, an NT ≥3.5 mm or a low PAPP-A level was significantly higher than those without a risk of these factors (Table 3). When none of these four risk factors were present, the prevalence of common and atypical abnormalities was 1.1 and 0.6%, respectively (Table 4); when one or more of these four risk factors were present, the corresponding prevalence was 11.5 and 1.7%, respectively (Table 4). In multivariable binary logistic regression analysis, all four of these risk factors were significant predictors of common abnormalities, while only a low PAPP_A and an NT ≥3.5 mm were significant predictors of atypical abnormalities, with adjusted ORs of 4.74 and 6.43, respectively (Table 5).

**Table 3:** Chromosome abnormalities by maternal age, risk of trisomy 21 (T21), nuchal translucency (NT), pregnancy associated plasma protein A (PAPP-A) and free β-human chorionic gonadotropin (fβ-hCG)

	Total pregnancies	Total abnormalities	T21,18 or 13 or SCA	Atypical abnormalities	*p*
High risk for T21 ≥ 1 in 250	1799	99	79 (4.4%)	20 (1.1%)	< 0.001*
High risk for 18 ≥ 1 in 180	5	4	3 (60.0%)	0 (0%)	
High risk for T21 and 18	51	34	33 (64.7%)	2 (3.9%)	
Risk of T21 > 1 in 100	793	111	97 (12.2%)	13 (1.6%)	< 0.001*
Risk of T21 < 1 in 100	1062	26	18 (1.7%)	9 (0.8%)	
Age ≥ 45	20	2	2 (10.0%)	0 (0%)	0.899
Age ≥ 35 < 45	1190	87	74 (6.2%)	14 (1.2%)	
Age < 35	645	48	39 (6.0%)	8 (1.2%)	
NT (mm) median (range)	1855	137	3.8 (1.1–12.2)	2.4 (1.4–12.6)	
NT MoM	1855	137	2.26 (0.68–7.19)	1.31 (0.82–7.15)	
NT ≥ 3.5 mm	216	65	55 (25.5%)	9 (4.2%)	< 0.001*
NT < 3.5 mm	1639	72	60 (3.7%)	13 (0.8%)	
PAPP-A MoM	1855	137	0.32 (0.05–2.32)	0.39 (0.09–1.64)	
Low PAPP-A	68	27	23 (33.8%)	5 (7.4%)	< 0.001*
Normal PAPP-A	1787	110	92 (5.1%)	17 (1.0%)	
fβ-hCG MOM	1855	137	1.44 (0.03–9.75)	1.60 (0.24–4.07)	
Low or high fβ-hCG	98	7	7 (7.1%)	0 (0%)	0.503
Normal fβ-hCG	1757	130	108 (6.1%)	22 (1.3%)	

*T* trisomy, *SCA* sex chromosome aneuploidies, *Atypical abnormalities* abnormalities other than trisomies 21, 18, and 13 and sex chromosome abnormalities, *MoM* multiple of medians, *low* < 0.2, *high* ≥5.0 MoM. N (%), median (range), as appropriate.

Chi-square test, *p < 0.05: significant

**Table 4:** Prevalence of common chromosome abnormalities [trisomies (T) 21, 18 and 13 and sex chromosome abnormalities] and atypical (or other) abnormalities in screen-positive pregnancies with or without any of the four risk factors, including a risk of T21 > 1 in 100,a nuchal translucency (NT) ≥ 3.5 mm, a low pregnancy associated plasma protein A (PAPP-A) < 0.2 multiple of medians, and a high risk for both trisomy 21 and trisomy 18

	Common abnormalities	Atypical abnormalities
No risk factors	1.1%	0.6%
One or more of the risk factors	11.5%	1.7%

**Table 5:** Prediction of common chromosome abnormalities [trisomies (T) 21, 18 and 13 and sex chromosome abnormalities] and atypical (or other) abnormalities by risk factors, including a risk of T21 > 1 in 100, a nuchal translucency (NT) ≥ 3.5 mm, a low pregnancy associated plasma protein A (PAPP-A) < 0.2 multiple of medians, and a high risk for both trisomy 21 and trisomy 18, after conventional screening

	Common abnormalities		Atypical abnormalities	
Factors	Unadjusted OR (95% CI)	Adjusted OR (95% CI)	Unadjusted OR (95% CI)	Adjusted OR (95% CI)
High risk for both T21 and T18	38.50 (20.80–71.25)*	12.14 (6.01–24.53)*	1.70 (0.22–12.87)	0.17 (0.02–1.67)
Risk of T21 > 1 in 100	8.08 (4.84–13.49)*	5.51 (3.26–9.31)*	2.37 (0.99–5.67)	1.44 (0.56–3.70)
NT ≥ 3.5 mm,	8.99 (6.03–13.42)*	4.05 (2.56–6.41)*	6.58 (2.81–15.42)*	6.43 (2.70–15.33)*
Low PAPP-A	9.42 (5.46–16.23)*	4.49 (2.16–9.35)*	6.14 (2.02–18.67)*	4.74 (1.49–15.01)*

*OR* odds ratio, *CI* confidence interval.

*statistically significant (*p* < 0.05), unadjusted OR after univariate analysis, adjusted OR after multivariable binary logistic regression

Of the total 1855 positive cFTS pregnancies, 935 (50.4%) did not have any of these four risk factors, and there were no significant differences in the prevalence of common or atypical or common abnormalities between women receiving cfDNA testing and those receiving IPD alone (1.1% vs. 1.0%, *p* = 0.873, 0% vs. 0.6%, *p* = 0.251). There were three atypical abnormalities, all of which were detected among the women who received IPD alone. The ages of these women were ≥ 35 years. These three chromosome abnormalities were mosaicism, including mos 47,XY,+r [18] dn [18] /46,XY [12], mos 47,XX,+mar dn [11] /46,XX [19], and 45,X [5]/46,XX[55]; all three pregnancies continued after proper counseling by a maternal fetal medicine team. The first patient had a pointed chin, mild malformed left ear and mild hearing loss, while the other two patients were phenotypically normal after birth. A repeated chromosomal analysis on the peripheral blood in these two patients showed normal results.

## Discussion

This study showed that the prevalence of common abnormalities increased with an increased cFTS risk, from 1.6% in women with T21 risk between 1 in 101 and 1 in 250 to 41.8% for women with risk ≥1 in 10. The prevalence of atypical abnormalities also increased with an increased cFTS risk in these women, from 0.9 to 3.6%, respectively. The prevalence of common abnormalities and that of atypical abnormalities was 1.1 and 0.6%, respectively, when none of these four risk factors (a risk of T21 > 1 in 100, a high risk of both T21 and T18, a PAPP-A < 0.2 MoM, and an increased NT) were present, and the prevalence was 11.5 and 1.7%, respectively, when one or more of these four risk factors were present. In the multivariable binary logistic regression analysis, all four of these risk factors and only the last two risk factors were significant predictors of common and atypical abnormalities, respectively.

These results are similar to the results of two large studies [12, 16]. In a study on the Danish population, the prevalence of atypical abnormalities increased in pregnancies with an increased fetal NT or cFTS risk, or those with a low PAPP-A [12]. The prevalence was 2.3, 1.6, and 4.2% in pregnancies with an NT in the >99th percentile, a cFTS risk> 1 in 100 and a low PAPP-A, respectively [12]. In a study on the Australian population, the prevalence of atypical abnormalities increased with an increased cFTS risk, and it was 4.6% in those pregnancies with a cFTS risk > 1 in 10 and 6.9% in pregnancies with a low PAPP-A [16].

Of all 22 pregnancies with atypical abnormalities, six, or 27.3%, did not have any of the abovementioned four factors, except for maternal age ≥ 35 in four of these six patients. These results are consistent with a previous study in which there were no such abnormal features in approximately 21% of pregnancies with atypical aneuploidies, except for a maternal age of 35 or above [13].

In contrast to previous studies [12, 16], we did not find that a greater maternal age and an abnormal level of fβ-hCG were significant risk factors for atypical abnormalities. This discrepancy may be due to the limitations of our present study, which include a small sample size, the exclusion of intermediate risk populations and a low percentage of samples tested with aCGH.

The present study was conducted at one large center over a five-year period in Chinese women. All except one abnormal cfDNA result were confirmed by IPD or after birth (Table 1). We used logistic regression analysis to assess the factors associated with common and atypical chromosome abnormalities. We did not present the sensitivity and specificity for each marker. The result of individual marker such as PAPP-A should be interpreted along with that of a cFTS. We do not have the full karyotype data on all pregnancies because some women underwent cfDNA testing instead of IPD. However, we did not find any newborns with significant chromosome abnormalities. Besides, there was no significant difference in the prevalence of atypical or common abnormalities between the one-year period before and the five-year period after the introduction of cfDNA testing (0.8% vs. 1.2%, *p* = 0.62; 5.8% vs. 6.2%, *p* = 0.8). One, or 4.5%, of the 22 atypical abnormalities was identified through an abnormal cfDNA testing result (Table 1). Recent studies have shown that cfDNA testing with advanced technologies can detect selected microdeletions or rare autosomal trisomies as well as common trisomies [17–21].

aCGH, which was self-financed, was performed in less than 10% of the IPD procedures, and the prevalence of atypical abnormalities in the present study was lower than that reported on routine CMA after high-risk cFTS (2.0–2.2%) [16, 22]. It is possible that a few atypical abnormalities in our cohort might have been missed. There is a need to increase the use of CMA, and further studies are required. In addition, cut-offs of cFTS vary among different countries. Variable and evolving platforms of cfDNA testing, offered by individual providers, may have a variable performance for SCA, mosaicism and other chromosome abnormalities [13, 23]. Of the 117 screen-positive women who declined further prenatal testing, seven had karyotyping of their newborns after birth of an abnormal phenotype (Table 1), and the remaining 110 did not have chromosome analysis because their babies were phenotypically normal. Among the screen-negative pregnancies, karyotyping was performed after birth only if abnormal phenotypes were found, and five babies with Down syndrome were diagnosed. All atypical abnormalities found were analyzed in the present study. Although some marker chromosomes and mosaic karyotypes are not accompanied by physiological problems in the affected children, prenatal prediction of their phenotype is difficult.

It is debatable how to best integrate cfDNA testing into current prenatal screening programs [24, 25]. A contingent approach of using cfDNA testing as a secondary screening tool following routine cFTS is a cost-effective approach [26, 27]. cFTS provides information on a priori risk for chromosome abnormalities [28] and can detect abnormalities other than trisomy 21 [29, 30]. At present, a cFTS report includes the risk of common trisomies but not that of SCA or atypical abnormalities. From the results of our present study, if none of these four risk factors (a risk of trisomy 21 > 1 in 100, a high risk of both trisomy 21 and trisomy 18, a low PAPP-A and an NT ≥3.5 mm) are present, which occurs in approximately half of cFTS-positive pregnancies, the prevalence of common and atypical abnormalities will be approximately 1.1 and 0.6% respectively, and the residual risk after a negative cfDNA testing will thus be small. If there is one or more of these risk factors, the prevalence of atypical abnormalities will range from 1.6 to 7.4% (Table 3), and IPD will be preferably offered as the first choice of treatment according to the current recommendations and evidence [12, 15, 16]. Because the prevalence of common abnormalities can be as high as 41.8% when the risk of trisomy 21 is > 1 in 10 by cFTS, there will also be a residual risk of missing common abnormalities after a negative cfDNA test if the fetal fraction and the precision of its measurement are low [27].

Using the abovementioned approach, we were able to detect approximately 72.7% (16/22) of atypical abnormalities in cFTS-positive pregnancies with 49.6% IPD. Offering IPD for an ultrasound-detected abnormality or an advanced maternal age ≥ 35 may help detect the remaining 27.3% [16]. More than half of the atypical abnormalities occurred in women with a cFTS below 1 in 300 and were found via ultrasound abnormalities [16]. Although approximately half of the prenatally detectable structural anomalies can be detected by a high-quality detailed scan at 12–13 weeks [31], some anomalies are only detectable in a mid-trimester scan. If CVS is performed in the first trimester for positive cFTS with one or more risk factors and if only karyotyping is performed, we suggest alerting the laboratory of the possibility of further testing by CMA, should anomalies be found subsequently. When deciding between cfDNA testing and IPD, patients use more than just the screening risk, and they may consider family values, finances and other issues. For some women who wish to maximize the detection of fetal atypical abnormalities, offering all high-risk cFTS pregnancies IPD with CMA is an option, but it comes with a procedure-related risk of miscarriage, albeit a small risk [22, 32]. For some women who wish to avoid an IPD, proper pre-test counseling before cfDNA testing should be provided to facilitate an informed choice [33]. Approximately one-fourth of these patients may not recognize the limited sensitivity of cfDNA testing, according to a previous local study [34]. Educating both healthcare professionals and patients is important [33].

## Conclusion

Similar to studies on Caucasians [12, 15, 16], our study identified the factors associated with common and atypical fetal abnormalities after cFTS. The factors can be used to estimate the prior risk of these abnormalities. The information may help the maternal decision regarding cfDNA testing versus IPD following a positive cFTS.

## Abbreviations

aCGH: Array comparative genomic hybridization
cFDNA: Cell-free DNA
cFTS: Combined first-trimester screening
CMA: Chromosome microarray
CVS: Chorionic villus sampling
fβ-hCG: Free β-human chorionic gonadotropin
IPD: Invasive prenatal diagnosis
NT: Nuchal translucency
PAPP-A: Pregnancy associated plasma protein-A
QEH: Queen Elizabeth Hospital
SCA: Sex chromosome aneuploidies
T: Trisomy
